# Impact of Order Set on Exocrine Pancreatic Insufficiency in Chronic Pancreatitis, Pancreatic Cancer, and Pancreatic Resection

**DOI:** 10.1016/j.gastha.2024.08.019

**Published:** 2024-08-30

**Authors:** Michael Ladna, Ishaan Madhok, Adnan Bhat, Nicole Ruiz, Jackson Brown, Jake Wilson, Peter Jiang, Robert Taylor, Mark Radetic, John George, Christopher Forsmark

**Affiliations:** 1 Department of Internal Medicine, University of Florida, Gainesville, Florida; 2 Division of Gastroenterology, Hepatology, and Nutrition, Department of Medicine, University of Florida, Gainesville, Florida

**Keywords:** Exocrine pancreatic insufficiency, Pancreatic enzyme replacement therapy, Chronic pancreatitis, Pancreatic cancer, Pancreatic resection

## Abstract

**Background and Aims:**

Enzyme insufficiency (EPI) is common in chronic pancreatitis (CP), pancreatic ductal adenocarcinoma (PDAC), and after pancreatic resection. 40%–50% of CP patients and 70%–80% of PDAC patients develop EPI. 1/3rd of these patients are prescribed Pancreatic enzyme replacement therapy (PERT), often at an inadequate dose, with evidence that this leads to increased morbidity and mortality. This study aimed to develop and implement an EPIC-based best practice alert (BPA) and smart set to improve the management of EPI.

**Methods:**

A retrospective analysis of all patients with International Classification of Diseases codes for EPI, CP, and PDAC or CPT code for pancreatic resection from Feb-2018 to Feb-2021. Appropriate use of PERT was defined as ≥ 40,000 units of lipase with each meal. The BPA and smart set were implemented into the electronic medical record in Feb-2020. The BPA fired if the patient was already on PERT or if an order for PERT was placed and directed the clinician to the smart set which provided PERT formulations each prefilled to the minimum therapeutic dose of 40,000 units of lipase.

**Results:**

A significant increase in the proportion of patients on minimum therapeutic dose of PERT from 61.9% to 72.9% (*P* ≤ .001). Ordering of pancreatic elastase, A1c, vitamin D, and dual X-ray absorptiometry increased from 20.4% to 29.9% (*P* < .001), 54.7%–62.1% (*P* = .001), 30.9%–48.1% (*P* < .001) and 10%–18% (*P* < .001), respectively. The BPA triggered a total of 30,838 times resulting in the smart being opened a total of 624 (2.02%) times over 24 months.

**Conclusion:**

The BPA and smart set were associated with an improvement in the diagnosis and management of EPI and related complications in CP, PDAC, and s/p pancreatic resection.

## Introduction

Pancreatic enzyme replacement therapy (PERT) is used to treat patients with pancreatic enzyme insufficiency (EPI). Other than cystic fibrosis (CF), EPI is a common complication in chronic pancreatitis (CP), pancreatic ductal adenocarcinoma (PDAC), and after pancreatic resection. 35%-50% of CP patients go on to develop EPI within 10–15 years of disease onset.^1^ Only a third of the CP population receive PERT and only a third of those get the minimum effective therapeutic dose of 40,000 units of lipase with each meal. The under-treatment of EPI is also prevalent in patients with PDAC and after pancreatic resection. Anywhere from 34%^2^ to 80% ^3^ of pancreatic resection patients go on to develop EPI postoperatively, with the majority being undertreated.^3^ Approximately 70% of patients with metastatic pancreatic cancer had malabsorption symptoms indicative of EPI yet only 21% of these patients were prescribed PERT.^4^^,^^5^ Several studies proposed that empiric prescription of PERT in the setting of metastatic pancreatic cancer^5^ and pancreatic resection^6^ was a reasonable therapeutic avenue. A meta-analysis did not show a difference in adverse events between patients who received PERT for treatment of EPI in the setting of CP and pancreatic resection vs those who did not receive PERT.^7^

EPI is an independent risk factor for cardiovascular events and mortality in CP^8^ and the absence of PERT was an independent risk factor associated with increased mortality.^6^ PERT offers mortality benefits for patients with unresectable pancreatic cancer^9^ which is especially important since >80% have unresectable disease.^10^^,^^11^ The degree of weight loss correlates with reduced survival in pancreatic cancer.^12^ PERT optimizes nutrition improving the tolerability and recovery from chemotherapy.^4^ This study aimed to assess deficits in the treatment of EPI in a large tertiary care center. A best practice alert (BPA) and smart set were designed and implemented into the electronic medical record (EMR) to improve the diagnosis and treatment of EPI and associated high-risk complications.

## Methodology

### Study Design

This retrospective cohort study was approved by the institution's institutional review board. All patients with an International Classification of Diseases (ICD-10) code diagnosis of EPI, CP, PDAC, or CPT code for pancreatic resection who were seen in either outpatient or inpatient setting from Feb 2018 until Feb 2021 were included. Patient medical record numbers were extracted from the institution’s integrated data repository and target diagnoses were confirmed via manual chart review. Data was collected via retrospective chart review. An initial chart review of data from Feb 2018 until Feb 2020 was done. Several deficits were identified in the diagnosis and treatment of EPI and associated high-risk complications. A smart set was developed with the intent to improve these deficits. In Feb 2020 the BPA and smart set were implemented into the institution's EMR. The initial design for the BPA was to fire for all patients with an ICD-10 diagnosis code for CP, PDAC, or EPI and current procedural terminology (CPT-10) code for pancreatic resection; however, this was not cleared by the BPA committee, and thus a compromise was reached for a more conservative BPA that would trigger for patients who were already on PERT or if PERT was being prescribed. The BPA would trigger for patients in seen in both the outpatient and inpatient clinical settings. The BPA would provide a link to the smart set. The smart set provided prespecified dosages of several of the most common PERT formulations and included orders for vitamin D level and supplementation, glycosylated hemoglobin, dual X-ray absorptiometry (DEXA) scan, fecal elastase, referrals to gastroenterology and endocrinology, and a smoking cessation program (refer to Figure A1 in Appendix). The BPA did not provide an automatic trigger for inpatient gastroenterology consultation. The decision for an inpatient gastroenterology consult was deferred to the inpatient health-care provider as EPI is a condition typically managed in the outpatient setting. There was concern for overburdening the inpatient gastroenterology team with consults that could be more appropriately addressed with a referral and clinic visit. A second retrospective chart review was done from Feb 2020 until Feb 2021. Patients aged less than 18, incarcerated individuals, patients with a diagnosis of CF, and patients who did not have a diagnosis of CP, PDAC, s/p pancreatic resection, or EPI on retrospective chart review from both the initial and subsequent data collections were excluded. There were educational presentations given to the internal medicine residents at their noon conference, the gastroenterology and hepatology fellows and faculty at GI grand rounds, and the entire medicine faculty at grand rounds on the initiation of BPA and smart set. An email was sent out to the entire medical department outlining the details of the BPA and smart set. There were no re-education or follow-up emails sent out after the implementation of the BPA and smart set.

### Study Outcome Measures

The primary aim of this study was to assess whether the BPA and smart set were associated with an increase in enzyme prescription and use of a minimum therapeutic dose of enzyme of 40,000u lipase with every meal. The secondary aims of this study were to assess for an increase in the ordering of pancreatic elastase, vitamin D, vitamin D supplementation, hemoglobin A1c, DEXA scans, diagnosis of metabolic bone disease, and monthly use of the smart set.

### Definitions

The diagnosis of CP was defined as either being made by a gastroenterologist at our institution, proven on histopathology, or the presence of classic findings of CP on cross-sectional imaging with appropriate symptoms. For cross-sectional imaging computed tomography, magnetic resonance cholangiopancreatography, or endoscopic ultrasound (EUS) were all deemed appropriate. For computed tomography the image findings for CP included pancreatic calcifications and pancreatic ductal dilatation. For magnetic resonance cholangiopancreatography the image findings for CP included reduced T1 signal intensity, while characteristic ductal changes included main pancreatic duct dilatation or irregularity, dilation of side branches, and the presence of at least 1 stricture. There are 4 parenchymal and 5 ductal criteria for CP diagnosis on EUS (refer to Table A11 in the appendix). Those who did not meet these diagnostic criteria via retrospective chart review despite having an ICD-10 diagnosis code for CP were excluded. The minimum therapeutic dose of PERT was set at 40,000u lipase with every meal. Following with a gastroenterologist was defined as at least 2 outpatient visits at our institution. A pancreatic elastase level of less than 200ug/g was defined as diagnostic for EPI. Due to nonspecific clinical signs, radiographic findings, and EUS criteria for EPI, patients who were not prescribed PERT were considered as not having a diagnosis of EPI.

### Statistical Analysis

All variable and outcome distributions were summarized as percentages for categorical variables and means with standard deviations for all continuous variables. The Shapiro-Wilk test was used to determine normality. The chi-square test was used to determine statistical significance with *P* < .05 indicating statistical significance. Odds ratios (ORs) were calculated via cross-tabulation. Statistical package for social sciences was used for the calculation of descriptive statistics. For pancreatic elastase levels less than or greater than a certain value, that value was set as the number for statistical analysis.

## Results

The integrated data repository identified a total of 3377 unique patient charts with the ICD-10 codes for CP, PDAC, and EPI or CPT-10 code for pancreatic resection from Feb 2018 to Feb 2021. Of these 1234 patients were excluded due to having a diagnosis of CF or no evidence of CP, PDAC, EPI, or pancreatic resection from chart review. A total of 2143 patients met the inclusion criteria for the study. The baseline analysis before BPA and smart set initiation from Feb 2018 to Feb 2020 identified 1464 patients (refer to Figure A2 in the appendix). Overall, 837 (57.2%) patients were prescribed PERT. Of those prescribed enzymes, 518 (61.9%) were on the minimum therapeutic dose of 40,000u lipase. Overall, 299 (20.4%) patients had a pancreatic elastase checked, 453 (30.9%) had a vitamin D level measured, 156 (10%) had a DEXA ordered, and 801 (54.7%) had a hgA1c checked (refer to Table 1).

**Table 1:** Demographic and Clinical Characteristics of all Patients Divided by Etiology for Initial Retrospective Data Collection From February 2018 to February 2020

Characteristic	Total population (n = 1464)	Chronic pancreatitis (n = 548)	Pancreatic cancer (n = 722)	Pancreatic resection (n = 251)	Not identified (n = 90)
Age (mean ± SD)	62.4 ± 14	56 ± 13.5	68.4 ± 10.8	62.7 ± 14.4	58.4 ± 16.4
Sex (% male)	54.9	54.1	57.2	118 (47.0)	48.9
Pancreatic elastase done (%)	299 (20.4)	174 (31.8)	37 (5.1)	40 (15.9)	64 (71.1)
Pancreatic elastase	n = 299	n = 174	n = 37	N = 40	n = 64
Low <200 (%)	230 (76.9)	124 (70.9)	30 (81.1)	29 (72.5)	55 (87.3)
Pancreatic elastase level					
>200 (%)	69 (23.2)	51 (29.3)	7 (18.9)	3 (12.5)	8 (12.7)
>100 < 200 (%)	52 (17.4)	26 (14.9)	2 (5.4)	5 (20.8)	19 (30.2)
>15 < 100 (%)	103 (34.6)	54 (31)	9 (24.3)	10 (41.7)	30 (47.6)
<15 (%)	74 (24.8)	43 (24.7)	19 (52.4)	6 (25)	6 (9.5)
Prescribed PERT (%)	837 (57.2)	371 (67.7)	320 (44.3)	204 (81.3)	65 (72.2)
Pancreatic enzyme	n = 837	n = 371	n = 320	N = 204	n = 65
Other (%)	12 (1.4)	9 (2.4)	1 (0.3)	1 (0.5)	1 (1.5)
Creon (%)	531 (63.4)	228 (61.5)	207 (64.7)	133 (65.2)	44 (67.7)
Zenpep (%)	264 (31.5)	122 (32.9)	107 (33.4)	58 (28.4)	15 (23.1)
Viokase (%)	30 (3.6)	12 (3.2)	5 (1.6)	12 (5.9)	5 (7.7)
Minimum dose 40,000u (%)	518 (61.9)	212 (57.1)	212 (66.3)	150 (73.5)	40 (61.5)
Viokase + PPI or H2 blocker (%)	n = 30	n = 12	n = 5	N = 12	n = 5
	21 (70)	8 (66.7)	4 (80)	8 (66.7)	5 (100)
Tobacco use active (%)	330 (22.5)	235 (42.9)	60 (8.3)	37 (14.7)	16 (17.8)
Tobacco use past (%)	894 (61.1)	396 (72.1)	392 (54.3)	134 (53.4)	51 (56.7)
Vitamin D deficiency (%)	n = 453	n = 246	n = 126	N = 66	n = 51
	291 (64.2)	162 (65.8)	76 (60.3)	45 (68.2)	32 (62.7)
Vit D supplement (%)	373 (25.5)	156 (28.5)	152 (21.1)	66 (26.3)	34 (37.8)
Vit D checked (%)	453 (30.9)	245 (44.7)	126 (17.4)	68 (27.1)	51 (56.7)
Vit D level (mean ± SD)	26.94 ± 14.7	26.8 ± 15.2	26.5 ± 13.7	26.7 ± 13.98	29.4 ± 16.7
Diagnosis of diabetes (%)	600 (41)	228 (41.8)	278 (38.5)	114 (45.4)	45 (50)
A1c checked (%)	801 (54.7)	375 (68.4)	306 (42.4)	148 (59.0)	57 (63.3)
A1c level (mean ± SD)	6.89 ± 2.8	6.8 ± 2.2	6.8 ± 1.8	6.9 ± 1.99	7.7 ± 7.6
Albumin low? <3.5 (%)	597 (40.8)	166 (32.2)	349 (48.3)	143 (57.0)	26 (28.9)
Albumin checked (%)	1370 (93.6)	515 (94.0)	671 (92.9)	242 (96.4)	82 (91.1)
Albumin level (mean ± SD)	3.5 ± 0.78	3.7 ± 0.8	3.3 ± 0.8	3.3 ± 0.73	3.7 ± 0.8
Metabolic bone disease					
Unknown (%)	1271 (86.8)	442 (80.6)	670 (92.8)	220 (87.6)	66 (73.3)
Known (%)	193 (13.2)	106 (19.4)	52 (7.2)	31 (12.4)	24 (26.7)
DEXA ordered (%)	156 (10)	80 (14.6)	36 (5)	21 (8.4)	22 (24.4)
GI following (%)	470 (32.1)	297 (54.2)	94 (13)	61 (24.3)	46 (51.1)
Insured (%)	1308 (89.3)	455 (82.0)	680 (94.2)	231 (92.0)	80 (88.9)
Insurance type					
Uninsured (%)	154 (10.5)	93 (17.0)	41 (5.7)	20 (8.0)	9 (10)
Medicaid (%)	195 (13.3)	126 (23)	44 (6.1)	22 (8.8)	12 (13.3)
Medicare (%)	769 (52.5)	210 (38.3)	467 (64.7)	136 (54.2)	44 (48.9)
Private (%)	340 (23.2)	119 (21.7)	170 (23.5)	73 (29.1)	25 (27.8)

PPI, proton pump inhibitor.

The second analysis after initiation of BPA and smart set from Feb 2020 to Feb 2021 identified 679 patients carrying an ICD-10 code for CP, EPI, PDAC, or a CPT-10 code for pancreatic resection (refer to Table 2). A statistically significant increase in the proportion of patients on a minimum therapeutic dose of PERT from 61.9% to 72.9% (OR 1.64, *P* < .001) was observed. Ordering of pancreatic elastase, A1c, vitamin D, and DEXA increased from 20.4% to 29.9% (OR 1.67, *P* < .001), 54.7%–62.1% (OR 1.36, *P* = .001), 30.9%–48.1% (OR 2.06, *P* < .001) and 10%–18% (OR 1.96, (*P* < .001), respectively after initiation of BPA and smart set. An increase in vitamin D supplementation from 25.5% to 33.4% (OR 1.45, *P* < .001) and an increase in the proportion of patients with known metabolic bone disease status from 13.2% to 23.3% (OR 1.99, *P* < .001) was also observed. There was no change in the proportion of patients on PERT of any dose with the pre and postorder set groups both having 57% of patients on PERT (refer to Table 3).

**Table 2:** Demographic and Clinical Characteristics of all Patients Divided by Etiology for Follow-Up Retrospective Data Collection From February 2020 to February 2021

Characteristic	Total population (n = 679)	Chronic pancreatitis (n = 344)	Pancreatic cancer (n = 280)	Pancreatic resection (n = 156)	Not identified (n = 53)
Age (mean ± SD)	62.6 ± 14.2	57.2 ± 14.3	69.0 ± 10.7	65.1 ± 14.3	62.6 ± 14.2
Sex (% male)	365 (53.8)	194 (56.4)	147 (52.5)	77 (49.4)	26 (49.0)
Pancreatic elastase done (%)	203 (29.9)	147 (42.7)	30 (10.7)	30 (19.2)	31 (58.5)
Pancreatic elastase	N = 203	N = 147	N = 30	N = 30	N = 31
Low <200 (%)	146 (71.9)	99 (67.3)	22 (73.3)	19 (63.3)	27 (87.1)
Pancreatic elastase level					
>200 (%)	57 (28.1)	48 (32.6)	8 (26.7)	11 (36.7)	4 (12.9)
>100 < 200 (%)	29 (14.3)	22 (15.0)	3 (10.0)	3 (10.0)	5 (16.1)
>15 < 100 (%)	65 (32.0)	41 (27.9)	6 (20.0)	5 (16.7)	18 (58.1)
<15 (%)	52 (25.6)	36 (24.5)	13 (43.3)	11 (36.7)	4 (12.9)
Pancreatic elastase level (mean ± SD)	152.1 ± 166.5	163.9 ± 170.5	126.5 ± 157.3	162.9 ± 173.0	132.0 ± 160.6
Prescribed PERT (%)	387 (57.0)	207 (60.2)	144 (51.4)	120 (76.9)	33 (62.3)
Pancreatic enzyme	N = 387	N = 207	N = 144	N = 120	N = 33
Other (%)	2 (0.5)	2 (0.97)	0 (0.0)	0 (0.0)	0 (0.0)
Creon (%)	312 (80.6)	159 (76.8)	121 (84.0)	101 (84.2)	28 (84.8)
Zenpep (%)	64 (16.5)	39 (18.8)	20 (13.9)	16 (13.3)	5 (15.2)
Viokase (%)	9 (2.3)	7 (3.4)	3 (2.1)	3 (2.5)	0
Minimum dose 40,000u (%)	281 (72.6)	148 (71.5)	106 (73.6)	97 (80.8)	23 (69.7)
Viokase + PPI or H2 blocker (%)	N = 9	N = 7	N = 3	N = 3	N = 0
	7 (77.8)	5 (71.4)	3 (100)	3 (100)	
Tobacco use active (%)	185 (27.2)				
Tobacco use prior (%)	253 (37.3)				
Vitamin D deficiency (%)	N = 326	N = 204	N = 88	N = 65	N = 37
	222 (68.1)	140 (68.6)	56 (63.6)	42 (64.6)	25 (67.6)
Vitamin D supplement (%)	225 (33.1)	105 (30.5)	97 (34.6)	61 (39.1)	23 (43.4)
Vitamin D checked (%)	326 (48.0)	204 (59.3)	88 (31.4)	65 (41.7)	37 (69.8)
Vitamin D level (mean ± SD)	31.4 ± 18.3	31.3 ± 19.4	30.5 ± 13.2	30.3 ± 16.6	34.4 ± 21.6
Diabetes (%)	284 (41.8)	155 (45.1)	109 (38.9)	72 (46.2)	21 (39.6)
A1c checked (%)	422 (62.2)	235 (68.3)	145 (51.8)	100 (64.1)	41 (77.4)
A1c level (mean ± SD)	6.7 ± 2.1	6.8 ± 2.3	6.7 ± 1.8	6.7 ± 1.8	6.3 ± 1.6
Albumin low? <3.5 (%)	N = 632	N = 317	N = 261	N = 155	N = 52
	165 (26.1)	64 (20.2)	91 (34.9)	46 (29.7)	9 (17.0)
Albumin checked (%)	632 (93.1)	317 (92.4)	261 (93.2)	155 (99.4)	51 (96.2)
Albumin level (mean ± SD)	3.6 ± 0.78	3.7 ± 0.76	3.3 ± 0.75	3.5 ± 0.79	3.6 ± 0.73
Metabolic bone disease					
Unknown (%)	521 (76.7)	260 (75.6)	227 (81.1)	116 (74.4)	34 (64.2)
Known (%)	158 (23.3)	84 (24.4)	523 (18.9)	40 (25.6)	19 (35.8)
DEXA ordered (%)	122 (18.0)	70 (20.4)	30 (10.7)	23 (14.7)	20 (37.7)
GI following (%)	225 (33.1)	166 (48.2)	36 (12.8)	38 (24.4)	24 (45.3)
Insured (%)	615 (90.6)	303 (88.1)	259 (92.5)	151 (96.8)	51 (96.2)
Insurance type					
Uninsured (%)	63 (9.3)	41 (11.9)	19 (6.8)	5 (3.2)	2 (3.8)
Medicaid (%)	87 (12.8)	58 (16.9)	20 (7.1)	15 (9.6)	8 (15.1)
Medicare (%)	356 (52.4)	165 (47.9)	164 (58.6)	90 (57.7)	30 (56.6)
Private (%)	173 (25.4)	80 (23.3)	77 (27.5)	46 (29.5)	13 (24.5)

PPI, proton pump inhibitor.

**Table 3:** Characteristics Prior to and After Best Practice Alert and Smart Set Implementation

Characteristic	Pre order set total (n = 1464)	Post order set total (n = 679)	Odds ratio (CI)	Pearson chi square
Prescribed pancreatic enzyme (%)	837 (57.2)	387 (57.0)	0.99 (0.82–1.19)	0.925
Minimum therapeutic dose (%)	518/837 (61.9)	282/387 (72.9)	1.64 (1.26–2.14)	<0.001^a^
Vitamin D checked (%)	453 (30.9)	326 (48.0)	2.06 (1.71–2.48)	<0.001^a^
Vitamin D supplementation (%)	373 (25.5)	225 (33.1)	1.45 (1.19–1.77)	<0.001^a^
A1c checked (%)	801 (54.7)	422 (62.1)	1.36 (1.13–1.64)	0.001^a^
DEXA ordered (%)	156 (10.0)	122 (18.0)	1.96 (1.51–2.54)	<0.001^a^
Metabolic bone disease status known (%)	193 (13.2)	158 (23.3)	1.99 (1.58–2.52)	<0.001^a^
Pancreatic elastase done (%)	299 (20.4)	203 (29.9)	1.67 (1.35–2.05)	<0.001^a^
GI following (%)	470 (32.1)	226 (33.3)	1.06 (0.87–1.29)	0.564

a Indicates a statistically significant result at ⍺ = 0.05.

The CP group had a statistically significant decrease in the percentage of patients on PERT of any dose from 67.9% to 60.2% (OR 0.72, *P* = .019); however, there was a significant increase in the proportion of patients on a minimum therapeutic dose of 40,000u lipase from 57% to 71.5% (OR 1.93, *P* < .001) (refer to Table 4). There was a trend towards more patients being on a minimum therapeutic dose of PERT in pancreatic cancer (66.3%–73.1%; OR 1.38, *P* = .141) and pancreatic resection (73.5%–81.8%, OR 1.46, *P* = .176) groups but these were not statistically significant.

**Table 4:** Characteristics Prior to and After Best Practice Alert and Smart Set Implementation for Chronic Pancreatitis

Characteristic	Pre order set total (n = 548)	Post order set total (n = 344)	Odds ratio (CI)	Pearson chi square
Prescribed pancreatic enzyme (%)	372 (67.9)	207 (60.2)	0.72 (0.54–0.95)	0.019^a^
Minimum therapeutic dose (%)	212/372 (57.0)	148/207 (71.5)	1.93 (1.34–2.78)	<0.001^a^
Vitamin D checked (%)	246 (44.9)	204 (59.3)	1.79 (1.36–2.35)	<0.001^a^
Vitamin D supplementation (%)	157 (28.6)	105 (30.5)	1.09 (0.82–1.47)	0.550
A1c checked (%)	376 (68.6)	235 (68.5)	0.99 (0.74–1.32)	0.975
DEXA ordered (%)	80 (14.6)	70 (20.4)	1.50 (1.05–2.14)	0.024^a^
Metabolic bone disease status known (%)	106 (19.3)	84 (24.4)	1.35 (0.97–1.86)	0.072
Pancreatic elastase done (%)	174 (31.8)	147 (42.7)	1.60 (1.21–2.12)	<0.001^a^
GI following (%)	296 (54.0)	167 (48.5)	0.80 (0.61–1.05)	0.112

a Indicates a statistically significant result at ⍺ = 0.05.

There was a statistically significant increase in the ordering of DEXA scans for the CP (80 [14.6%] vs 70 [20.4%] OR 1.50; *P* = .024), PDAC (36 [5.0%] vs 30 [10.7%], OR 2.29; *P* < .001), and pancreatic resection (21 [8.4%] vs 23 [14.7%], OR 1.89; *P* = .044) groups. There was a statistically significant increase in vitamin D being ordered for the CP (246 [44.9%] vs 204 [59.3%], OR 1.79; *P* < .001), PDAC (126 [17.5%] vs 88 [31.4%], OR 2.17; *P* < .001), and pancreatic resection (68 [27.1] vs 65 [41.7], OR 1.92; *P* = .002) groups. The PDAC and pancreatic resection groups had a significant increase in patients on vitamin D supplementation and known metabolic bone disease status while the CP (174 [31.8%] vs 147 [42.7%], OR 1.6; *P* < .001) and PDAC (37 [5.1%] vs 30 [10.7%], OR 2.22; *P* < .001) groups had a significant increase in ordering of fecal pancreatic elastase (refer to Table 5 and 6).

**Table 5:** Characteristics Prior to and After Best Practice Alert and Smart Set Implementation for Pancreatic Cancer

Characteristic	Pre order set total (n = 722)	Post order set total (n = 280)	Odds ratio (CI)	Pearson chi square
Prescribed pancreatic enzyme (%)	320 (44.3)	144 (51.4)	1.33 (1.01–1.75)	0.043^a^
Minimum therapeutic dose (%)	212/320 (66.3)	106/144 (73.6)	1.38 (0.897–2.14)	0.141
Vitamin D checked (%)	126 (17.5)	88 (31.4)	2.17 (1.58–2.98)	<0.001^a^
Vitamin D supplementation (%)	152 (21.1)	97 (34.6)	1.99 (1.47–2.69)	<0.001^a^
A1c checked (%)	306 (42.4)	145 (51.8)	1.46 (1.12–1.93)	0.007^a^
DEXA ordered (%)	36 (5.0)	30 (10.7)	2.29 (1.38–3.79)	0.001^a^
Metabolic bone disease status known (%)	52 (7.2)	53 (18.9)	3.01 (1.99–4.54)	<0.001^a^
Pancreatic elastase done (%)	37 (5.1)	30 (10.7)	2.22 (1.34–3.67)	0.001^a^
GI following (%)	94 (13.0)	35 (12.6)	0.96 (0.64–1.46)	0.856

a Indicates a statistically significant result at ⍺ = 0.05.

**Table 6:** Characteristics Prior to and After Best Practice Alert and Smart Set Implementation for Pancreatic Resection

Characteristic	Pre order set total (n = 251)	Post order set total (n = 156)	Odds ratio (CI)	Pearson chi square
Prescribed pancreatic enzyme (%)	204 (81.3)	120 (76.9)	0.77 (0.47–1.25)	0.289
Minimum therapeutic dose (%)	150/204 (73.5)	97/120 (80.2)	1.46 (0.84–2.51)	0.176
Vitamin D checked (%)	68 (27.1)	65 (41.7)	1.92 (1.26–2.93)	0.002^a^
Vitamin D supplementation (%)	66 (26.3)	61 (39.4)	1.82 (1.19–2.79)	0.006^a^
A1c checked (%)	148 (59.0)	100 (64.1)	1.24 (0.82–1.88)	0.302
DEXA ordered (%)	21 (8.4)	23 (14.7)	1.89 (1.01–3.55)	0.044^a^
Metabolic bone disease status known (%)	31 (12.4)	40 (25.6)	2.45 (1.46–4.12)	<0.001^a^
Pancreatic elastase done (%)	40 (15.9)	30 (19.2)	1.26 (0.74–2.12)	0.392
GI following (%)	62 (24.7)	38 (24.3)	0.96 (0.60–1.54)	0.878

a Indicates a statistically significant result at ⍺ = 0.05.

An analysis of patients who did not have EPI (defined as patients who were not prescribed PERT) showed a statistically significant increase in the ordering of pancreatic elastase (66 [10.5%] vs 54 [18.5%], OR 1.929; *P* < .001), vitamin D level (141 [22.5%] vs 114 [38.9%], OR 2.195; *P* < .001), vitamin D supplementation (117 [18.7%] vs 75 [25.7%], OR 1.507; *P* = .015), DEXA scans (48 [7.7%} vs 39 [13.3%], OR 1.852; *P* = .006), as well as an increase in the diagnosis of metabolic bone disease (50 [8.0%] vs 38 [13.0%], OR 1.720; *P* = .016) after implementation of BPA and smart set. There was no difference in the ordering of A1c or the proportion of patients who were established with a gastroenterologist at our institution (refer to Table A7 in the appendix).

There was no improvement in the proportion of patients who were followed in a gastroenterology clinic after the implementation of the BPA and smart set (470 [32.1%] vs 226 [33.3%]; *P* = .564). However, for those patients following with a gastroenterologist, there was a statistically significant increase in the proportion of patients on PERT and minimum therapeutic dose of PERT as well as the ordering of pancreatic elastase, vitamin D level, vitamin D supplementation, DEXA scans, and diagnosis of metabolic bone disease. This was true for both before and after the implementation of the BPA and smart set (refer to Tables A8 and A9 in the appendix).

The BPA was triggered a total of 30,838 times over the 24 months immediately after its implementation. Only 624 (2.02%) of these triggers resulted in the smart set being opened (refer to Table A10 in the Appendix). There was a slight decrease in the monthly use of the smart set over a period of 24 months after initial implementation with a slope of negative 0.2016 (refer to Figure A3 in the appendix).

## Discussion

This study identified several deficits, even in a tertiary care center and a pancreatic center of excellence, in the diagnosis and management of EPI. EPI can be difficult to diagnose given nonspecific symptoms and diagnostic tests that at best have moderate sensitivity and specificity with high false negative and false positive rates. Fecal elastase was found to have a pooled sensitivity of 0.77 (95% CI, 0.58–0.89) and specificity of 0.88 (95% CI, 0.78–0.93).^13^ In patients with a low pretest probability of EPI, the fecal elastase had a false negative rate of 1.1% and a false positive rate of 11% indicating it has a high yield in ruling out EPI but not in the detection of EPI. In contrast, for patients with a high pretest probability of EPI, the false negative rate increased to 10%.^13^ This means that a negative fecal elastase test in a patient with high suspicion for EPI is insufficient in ruling out the diagnosis requiring further investigation.

The percentage of patients on a minimum therapeutic dose of PERT increased from 61.9% to 72.9% (OR 1.64; *P* < .001). Although this was a statistically significant increase, 17.1% of patients were still on a subtherapeutic dose after the BPA and smart set were initiated. These continued deficits were likely explained by the constrained period of 12 months in which the BPA and smart set were active before the second round of data collection. In addition, there remain significant difficulties with patients of lower socioeconomic status affording PERT since the cost is approximately $1.44 to $13.89 per unit (range of lipase in a single unit ranging from 3,000 to 40,000 USP) with an expected out-of-pocket cost averaging $999 (range, $853 to $1536) for a 30-day supply of optimally dosed PERT.^14^ This renders PERT unaffordable for many patients and limits the physician's ability to prescribe PERT or increase the dose to a minimum therapeutic dose of 40,000 units of lipase. This issue is further compounded since some patients will require a larger dose than 40,000 units of lipase to experience a clinical benefit.

There was a statistically insignificant decrease in the proportion of CP patients on PERT. This decrease is not as significant as it first appears since prior studies have shown that overall, 35%–50% ^1^ of CP patients go on to develop EPI. Thus having between 60%-70% of all CP patients on PERT may reflect that the majority of CP patients who develop EPI are receiving PERT at our institution. Although the prescription of PERT for the treatment of EPI in PDAC was better at our institution compared to others (Landers et al reported 21% of metastatic pancreatic cancer being prescribed PERT^4^^,^^5^) this likely still represented a significant undertreatment and underdiagnosis in this population since studies have reported that 66%–92% of patients with inoperable PDAC went on to develop EPI.^15^ The minority of PDAC patients who are diagnosed early and do not have metastatic disease are often taken to surgery. The proportion of pancreatic resection patients on PERT did not have a statistically significant increase after initiation of BPA and smart set. However, as with CP patients given that up to 80% of pancreatic patients go on to develop EPI,^3^ this population was likely adequately treated at our center. However, it could be argued that the prevalence of EPI in current literature could be an underestimate given the difficulty in establishing a diagnosis of mild EPI.^2^ Based on our data PDAC patients were the most undertreated compared to CP and pancreatic resection.

The increase in ordering of Hgb A1c, vitamin D level, vitamin D supplementation, and DEXA scans can have significant positive impacts on both patient morbidity and mortality. Effective and regular screening for diabetes can result in an earlier diagnosis and initiation of treatment with goal of slowing progression to complications such as diabetic retinopathy, nephropathy, and neuropathy. Screening and earlier detection of metabolic bone disease via DEXA scans also offer significant benefits. Hip fractures have a 1-year mortality rate of 20%–33%^16^^,^^17^ and only about one-third of patients regain their prefracture level of physical functioning.^18^ Vertebra fractures are also devastating with a 1-year survival rate of 72% and 5-year survival of only 28%.^17^ Osteopenia was associated with increased nontrauma-related mortality with each standard deviation decrease in proximal radius bone mineral density associated with a 1.19-fold increase in mortality.^19^ Diminished bone mineral density was strongly associated with deaths from strokes (relative risk = 1.74) and in fact, most deaths in women with osteopenia were unrelated to fractures.^19^ Vitamin D deficiency is a risk factor for fractures in the elderly.^20^ Treatment of osteoporosis after a hip fracture with zoledronic acid was associated with a 35% risk reduction of any new clinical fracture and a 28% risk reduction in deaths of any cause.^21^

Over a period of 24 months, there was a consistent use of the smart set without the need for re-education. Novel smart sets should be implemented alongside a BPA. The automatic trigger prevents the need to regularly re-educate health-care providers on the existence of the smart set. A major drawback of our BPA is that it fired for all patients on PERT not just those on subtherapeutic doses. This can lead to fatigue and frustration amongst health-care providers due to additional clicking while interfacing with the EMR. Our BPA also missed all patients with the targeted high-risk diagnoses who either were not already on PERT or for whom PERT was not prescribed which was a limitation placed on the BPA by the administration to prevent excessive triggering. This limitation likely explains why there was no change in the proportion of patients on PERT of any dose after initiation of the BPA and smart set. Future BPA should include this sizeable patient population but at the same time exclude patients already on minimum therapeutic dose of PERT to prevent excess unnecessary triggering which leads to alarm fatigue.

Besides CP, PDAC, pancreatic resection, and CF, other diseases associated with EPI include diabetes mellitus, small intestinal bacterial overgrowth, celiac disease, inflammatory bowel disease due to loss of intestinal brush border proteins, hemochromatosis, and gastrointestinal surgeries other than pancreatic resections due to inappropriate mixing of pancreatic juices with ingested food.^15^ Further studies are required to determine the prevalence of EPI in these less-recognized high-risk populations and whether regular screening is appropriate.

There were several limitations in this study. Firstly, fecal elastase was the only pancreatic function test assessed in this study. Although a flawed diagnostic test it does offer several advantages that resulted in it being chosen. Other noninvasive tests include fecal chymotrypsin and 72-hour fecal fat estimation. The fecal chymotrypsin assay is limited because chymotrypsin is prone to proteolytic degradation and the assay is unable to differentiate the exogenous chymotrypsin found in PERT formulations.^22^ Quantitative (72-hour) fecal fat estimation is considered the gold standard among the indirect pancreatic function tests; however, this test is cumbersome and generally not tolerated well by patients.^23^ Unlike fecal chymotrypsin, pancreatic elastase-1 is highly stable throughout the gastrointestinal tract due to a lack of proteolytic degradation and can assess pancreatic function even if the patient is already on PERT.^24^ Fecal elastase is relatively inexpensive, covered by most health insurance providers, and requires <1g of stool.^25^ Fecal pancreatic elastase works best as a screening test in patients with known high-risk pathology such as CP but equivocal clinical signs or radiographic findings for EPI. Once a diagnosis of EPI is made and PERT initiated the test is of limited utility unless it is used to help disprove a questionable EPI diagnosis. Although there was an increase in the ordering of fecal elastase in patients without EPI to 18.5% this is a small percentage and further efforts are required to implement the regular use of elastase as a screening tool in this patient population.

The initial education carried out via email and PowerPoint presentations may have contributed to the improvements seen in the management of EPI separate from the BPA and smart set. We were unable to determine the effect these educational and awareness initiatives had on the results presented in this project. The initial rendition of the smart set did not include vitamin A or vitamin E lab draws. Since vitamin D deficiency is common even amongst patients without EPI, vitamins A and E are superior surrogates for malabsorption. Smart sets implemented at other centers should strongly consider the inclusion of vitamins A and E.

The location of the pancreatic malignancy or the portion of the pancreas that was resected was not documented. Cancer or resection to the head of the pancreas likely predisposed patients to the development of EPI over the tail of the pancreas. However, since most pancreatic cancer occurs in the head of the pancreas this is less of a limitation than initially conceived. Compared to other forms of pancreatic resection the Whipple procedure was more significantly associated with the development of EPI.^26^ In addition, the stage of malignancy was not differentiated. 66%–92% of patients with inoperable pancreatic cancer went on to develop EPI.^15^ Early-stage pancreatic malignancy is less often associated with the development of EPI; however, these patients are often taken to surgery for pancreatic resection, and thus still go on to have a significant risk of developing EPI.

Following with a gastroenterologist resulted in improved adherence to EPI clinical guidelines in patients with CP, PDAC, and postpancreatic resection. Despite the smart set including a referral to gastroenterology, we did not appreciate an increase in the proportion of patients following with GI. There may be several explanations for this. Firstly, the second retrospective data review utilized 12 months of data immediately after the implementation of BPA and smart set. Gastroenterology clinics are typically quite busy and it could take several months from the input of a referral order until the patient is seen by a gastroenterologist in clinic. In addition, the criteria set by our study required a minimum of 2 clinic visits to qualify as following with GI. The timeline from the referral order to a first clinic visit and then a second clinic visit could take several months up to a year and as such it is likely that patients who had established with GI but were not seen for a follow-up appointment yet may have been missed in our retrospective chart review. Due to not having reliable access to outside medical records patients who were already established with a gastroenterologist outside of our institution were also missed.

In conclusion, a BPA and smart set provide a durable low-cost mechanism to improve practice patterns regarding the diagnosis and treatment of EPI in CP, PDAC, and status post pancreatic resection. EPI is underdiagnosed and undertreated in patients with CP, PDAC, and pancreatic resection. There is still great opportunity for improvement in the management of EPI especially in the realm of diagnosis early in the course of the disease. Further research should focus on optimizing diagnostic tools and implementation of regular screening protocols for EPI in high-risk populations such as those with CP, PDAC, and after pancreatic resection.
