# Supplementary material for: Impact of Order Set on Exocrine Pancreatic Insufficiency in Chronic Pancreatitis, Pancreatic Cancer, and Pancreatic Resection
Source: Gastro Hep Adv. 2024 Aug 30;4(1):100541. doi: 10.1016/j.gastha.2024.08.019 (PMC11713485; doi:10.1016/j.gastha.2024.08.019)
Supplement: Appendix [file mmc1.pdf]

APPENDIX

FIGURE 1: Smart Set as seen in the electronic medical records (EMR)

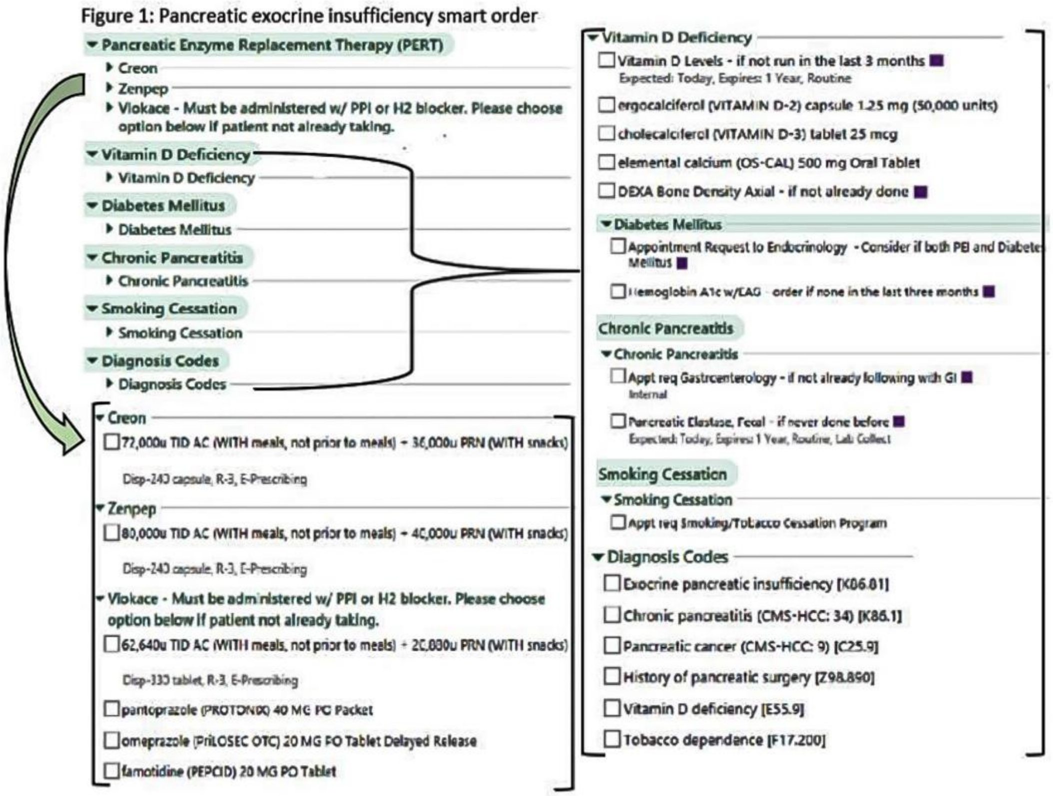

FIGURE 2: Diagram of patient flow through the study

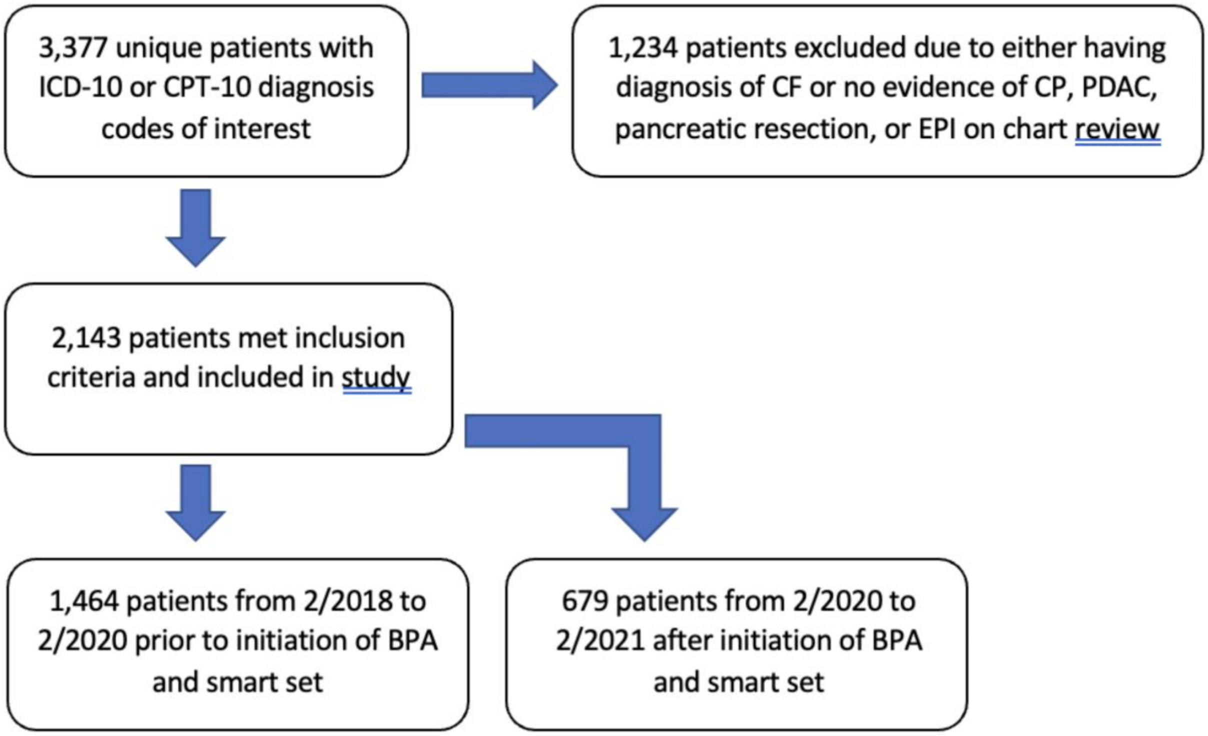

FIGURE 3: Smart set openings per month over 24 months since activation of BPA and smart set

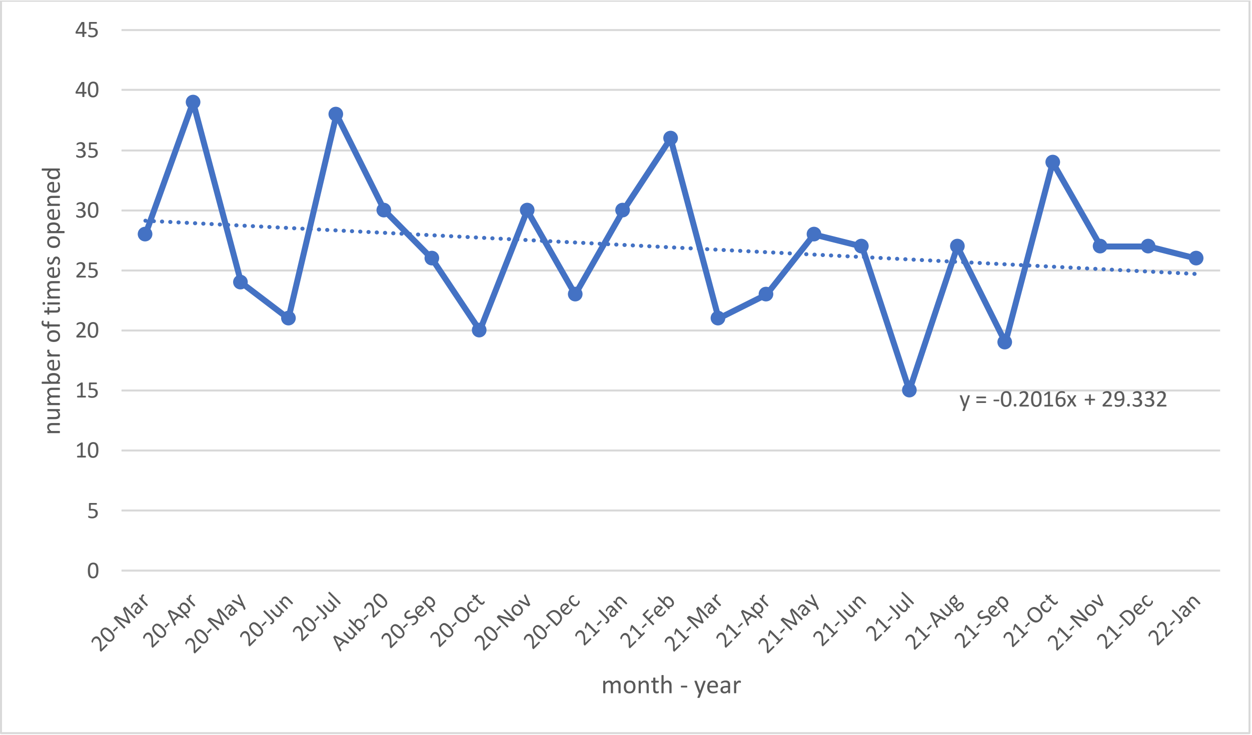

TABLE 7: Characteristics before and after BPA and smart set implementation for patients without EPI (defined as patients not prescribed any form of PERT)

	Pre order set (n=627)	Post order set (n=292)	Odds ratio (CI)	P value
Vitamin D supplementation (%)	117 (18.7%)	75 (25.7%)	1.507 (1.082 – 2.097)	0.015*
Vitamin D checked (%)	141 (22.5%)	114 (38.9%)	2.195 (1.625 – 2.965)	<0.001*
A1c checked (%)	322 (51.4%)	167 (57.0%)	1.255 (0.949 – 1.660)	0.110
DEXA ordered (%)	48 (7.7%)	39 (13.3%)	1.852 (1.184 – 2.898)	0.006*
Pancreatic elastase checked (%)	66 (10.5%)	54 (18.5%)	1.929 (1.305 – 2.849)	<0.001*
Metabolic bone disease (%)	50 (8.0%)	38 (13.0%)	1.720 (1.100 – 2.688)	0.016*
Following with gastroenterology (%)	148 (23.6%)	56 (19.2%)	0.768 (0.544 – 1.084)	0.133

**P <0.05 indicates statistically significant result*

TABLE 8: Characteristics between patients following with and not following with Gastroenterology before BPA and smart set implementation.

	Not following with GI (n=994)	Following with GI (n=470)	Odds ratio (CI)	P-value
Pancreatic elastase checked (%)	105 (10.6%)	194 (41.3%)	5.951 (4.529 - 7.821)	<0.001*
Prescribed PERT (%)	515 (51.8%)	322 (68.5%)	2.024 (1.606 - 2.549)	<0.001*
Minimum therapeutic dose of PERT (%)	N=515 300 (58.3%)	N=322 218 (67.7%)	1.502 (1.122 - 2.012)	0.006*
DEXA ordered (%)	48 (4.8%)	99 (21.1%)	5.259 (3.651 - 7.576)	<0.001*
A1c checked (%)	485 (48.8%)	316 (67.2%)	2.153 (1.712 - 2.708)	<0.001*
Vitamin D supplementation (%)	210 (21.1%)	163 (34.7%)	1.982 (1.554 - 2.529)	<0.001*
Vitamin D checked (%)	218 (21.9%)	235 (50.0%)	3.560 (2.814 - 4.503)	<0.001*
Metabolic bone disease (%)	87 (8.8%)	106 (22.6%)	3.036 (2.230 - 4.133)	<0.001*
Diagnosis of diabetes (%)	388 (39.0%)	212 (45.1%)	1.283 (1.028 - 1.602)	0.027*

**P <0.05 indicates statistically significant result*

TABLE 9: Characteristics between patients following with and not following with Gastroenterology after BPA and smart set implementation.

	Not following with GI (n=450)	Following with GI (n=225)	Odds ratio (CI)	P-value
Pancreatic elastase checked (%)	62 (13.8%)	137 (60.9%)	9.718 (6.653 - 14.194)	<0.001*
Prescribed PERT (%)	215 (47.8%)	169 (75.1%)	3.328 (2.335 - 4.743)	<0.001*
Minimum therapeutic dose of PERT (%)	N=215<br>141 (65.6%)	N=169<br>138 (82.1%)	2.414 (1.487 - 3.920)	<0.001*
DEXA ordered (%)	37 (8.2%)	83 (36.9%)	6.509 (4.226 - 10.024)	<0.001*
A1c checked (%)	250 (55.7%)	167 (74.2%)	2.292 (1.612 - 3.259)	<0.001*
Vitamin D supplementation (%)	131 (29.1%)	91 (40.4%)	1.685 (1.203 - 2.361)	0.002*
Vitamin D checked (%)	158 (35.1%)	164 (72.9%)	4.969 (3.495 - 7.065)	<0.001*
Metabolic bone disease (%)	59 (13.1%)	63 (28.0%)	2.577 (1.728 - 3.843)	<0.001*
Diagnosis of diabetes (%)	178 (39.6%)	107 (47.6%)	1.386 (1.003 - 1.913)	0.047*

**P <0.05 indicates statistically significant result*

TABLE 10: Proportion of BPA triggering that lead to the opening of the smart set (highlighted in green) within the EMR.

ACTION TAKEN	COUNT (percent)
Accept BPA BUT smart set not opened (%)	946 (3.07)
Acknowledge/Override warning (%)	603 (1.96)
Cancelled/deferred BPA (%)	104 (0.34)
blank/unspecified (%)	28,561 (92.62)
smart set opened (%)	**624 (2.02)**
**TOTAL**	**30,838**

TABLE 11: criteria for diagnosis of CP using EUS

**Parenchymal criteria**	**Ductal criteria**
1) Lobularity<br>2) Cysts<br>3) Hyperechoic foci<br>4) Hyperechoic strands	1) Dilation<br>2) Irregularity<br>3) Calcifications and stones<br>4) Echogenic ductal walls<br>5) Side Branches
